# Assessing volatile organic compound level in selected workplaces of Kathmandu Valley

**DOI:** 10.1016/j.heliyon.2021.e08262

**Published:** 2021-10-27

**Authors:** Madhav Kharel, Surendra Chalise, Baburam Chalise, Khaga Raj Sharma, Deepak Gyawali, Hari Paudyal, Bhanu Bhakta Neupane

**Affiliations:** a Central Department of Chemistry, Tribhuvan University, Kathmandu, Nepal; b Ministry of Forests and Environment, Department of Environment, Government of Nepal, Nepal

**Keywords:** Air pollution, Volatile organic compounds, Indoor air quality, Workplace safety, VOC sensor

## Abstract

Volatile organic compounds (VOCs) are one of the major contributors to poor indoor air quality. Due to advancements in sensor technologies, continuous if not regular monitoring total VOC (TVOC) and or some specific VOC in potential high risk workplaces is possible even in resource limited settings. In this study, we implemented a portable VOC sensor to measure concentration of TVOC and formaldehyde (HCHO) in six types of potential high risk workplaces (n = 56 sites) of Katmandu Valley. For comparison, concentration was also measured in immediate surroundings (n = 56) of all the sites. To get preliminary information on safety practices, a survey study was also conducted. The mean TVOC and HCHO concentration in the sites ranged from 1.5‒8 mg/m^3^ and <0.01–5.5 mg/m^3^, respectively. The indoor: outdoor TVOC and HCHO ratio (I/O) was found to be significantly higher (I/O > 1.5 and p < 0.05) in 34 (~61%) and 47 sites (∼84%), respectively. A strong positive correlation between HCHO and TVOC concentration was observed in furniture industry (R = 0.91) and metal workshops (R = 0.98). Interestingly, we found TVOC and HCHO concentration higher than WHO safe limit in ∼64% and ∼32% sites, respectively. A rough estimate of chronic daily intake (CDI) of formaldehyde showed that CDI is higher than WHO limit in four sites. These findings suggested that indoor air quality in the significant number of the workplaces is poor and possible measures should be taken to minimize the exposure.

## 1 Introduction

Air quality has been one of major health concerns globally. A most recent estimate shows that the combined effect of outdoor and indoor air pollution leads to around seven million early deaths annually [1,2]. In 2016, the outdoor and indoor air pollution related deaths were 4.2 and 3.8 million, respectively [3]. Interestingly, most recent WHO data showed that around 90% global people breathe low quality air [2]. The increased emission of primary air pollutants such as particulate matter, carbon monoxide, volatile organic compounds, polycyclic aromatic hydrocarbons, nitrogen oxides, sulfur dioxide, and ozone has led to poor air quality in many cities across the globe [1,3]. The poor air quality and health effect has been a subject of considerable interest to scientific community and public. Several groups across the globe are actively exploring different aspects of air pollution such as source identification, short and long term health effects, and mitigation strategies [4, 5, 6, 7, 8, 9, 10].

Volatile organic compounds (VOCs) are the chemicals having low boiling point and capable of producing photochemical oxidants in presence of other atmospheric precursors and sunlight [11]. They are known to release in the environment via various sources such as burning of fuels, industry, and personal care products. Several studies have shown that exposure to airborne VOCs leads to short and long term health effects such as nose and throat discomfort, allergy, nausea, headache, visual disorders and memory impairment, damage to liver and kidney, ashthma, and even to cancer [12]. The health effect of a VOC depends on its chemical nature, concentration and length of exposure. Formaldehyde is one of the most abundant VOCs in indoor workplaces. An elevated level of formaldehyde can cause several short and long term health effects such as burning sensation, nausea, skin irritation and even cancer [13,14]. This brings the importance of determination of level of individual and or total VOCs in both outdoor and indoor environments. Several studies have shown an elevated level of VOC in or near workplaces such as printing press dyeing industry and school zones [15, 16, 17, 18].

Identification and quantification of individual VOC in indoor and outdoor environments can be made by instrumental techniques such as GC and GC‒MS and its variants. Level of VOC in atmosphere is generally low, so before quantification the VOC of interest is required to be extracted and/or concentrated by using various techniques. The common techniques available for extraction and concentration can be broadly classified into two categories [19]; a) whole air collection in specially designed canisters, b) solid sorbent techniques. In the former one, the total air is directly collected in an especially designed and deactivated canister and concentrated by cryo trap or focusing in a GC instrument. In the later method, the VOC of interest is extracted and concentrated by active or passive sampling methods using a specially designed tube (for example diffusive sampler) containing adsorbents such as activated carbon or Tenax. The VOC of interest is desorbed chemically using solvents such as CS_2_ and methanol or thermal desorption methods. Although chromatographic methods provide information of individual VOC, these methods usually require dedicated separation instruments, trained manpower, and time‒consuming. In recent years low cost GC instruments are being explored for determination of few VOCs in real time [20,21], the traditional GC instruments are difficult to use in resource limited environments and in potential high VOC workplaces where continuous if not frequent monitoring is required.

Due to advancement in sensor technologies, direct monitoring of total VOC (TVOC) in different environments is made possible [22]. To date, chemical sensors having variable limit of detection, concentration range, and response time are explored, and among them photo ionization detectors (PID), metal oxide semiconductors (MOS), non-dispersive infrared (NDIR), amperometric, and thermal (Pellistor) are some of the promising low cost sensors [22, 23, 24, 25]. Majority of these sensors, cannot provide concentration of specific VOCs but offer real time monitoring or screening of total VOCs. They are being used in assessing indoor air quality in several environments such as printing press [15], university offices and rooms [26, 27, 28], nail salons [29], food industry [30] of different countries. These studies have reported an elevated level of TVOC and or individual VOCs in the workplaces. Similar studies in the potential high risk workplaces of Nepal is not explored.

In this study, we measured TVOC and HCHO concentration in six different workplaces (n = 56 sites) of Kathmandu Valley using a commercial field based VOC sensor. For comparison, measurement was also made in immediate surroundings (n = 56) and high VOC spots were identified in each indoor sites. A survey study was conducted to get a preliminary information on safety practices in the workplaces. We also made a rough estimate on chronic daily uptake of HCHO in the workplaces. Finally, the measured and estimated concentration of both TVOC and HCHO was also compared with the WHO recommended limit.

## 2 Materials and methods

### 2.1 Survey study

A survey questionnaire was designed to get information on the employment history, safety practices, and work related health issues. The questions were prepared in printed form. The survey questionnaire was reviewed and approved by head of the research committee of Central Department of Chemistry, Tribhuvan University. Informed consent was also obtained from all the participants before data collection. Depending on level of literacy of the workers, the information was collected by direct participation or via interviews. The survey questionnaires used in this study is provided in supporting information.

### 2.2 Determination of TVOC and HCHO

A handheld TVOC/HCHO meter (Extech, VFM200, USA) was used for the measurement total VOC and formaldehyde in the selected indoor and outdoor sites. The meter had two in‒built sensors; front electrochemical type HCHO and rear MOS type TVOC sensors. These sensors had large surface area allowing direct measurement VOC in the air diffused on the sensor surface. The reported linear working range for TVOC and HCHO sensors were 0.01–10 ppm and 0.01–5 ppm, respectively. Limit of detection, full scale (FS) accuracy, and response time were 0.01 ppm, ±5%FS, and ≤2 s, respectively. The sensors had broad working range of humidity (15–95%) and temperature (0–40 °C). Before measurement, the sensors were factory calibrated using 10 ppm formaldehyde gas and zero calibrated using 90% N_2_ and 10% O_2_ mixture.

**Figure 1:**
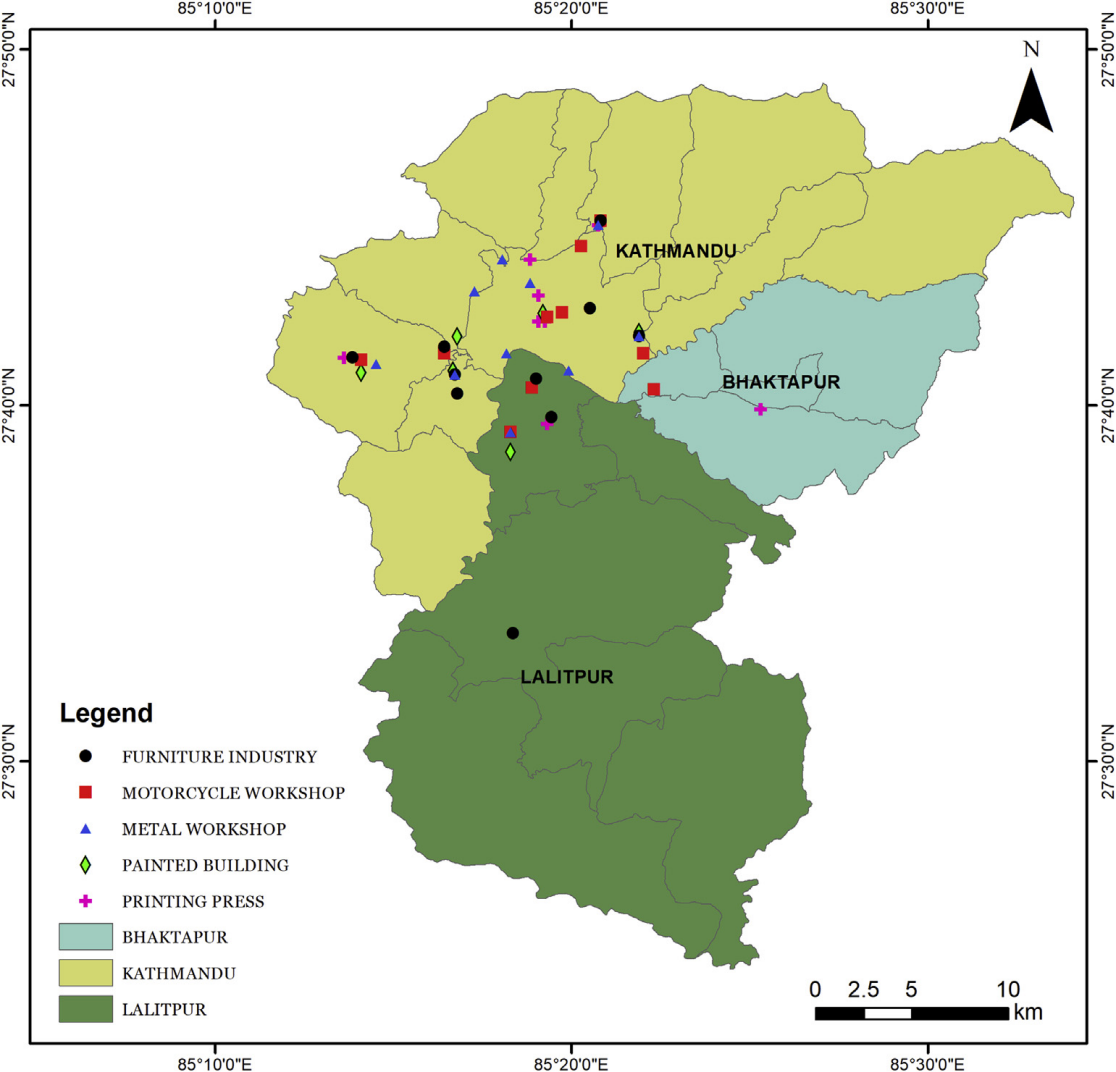
Study sites. The study areas included were printing presses (n = 10), furniture industries (n = 10), newly painted buildings (n = 6), metal workshops (n = 10), motor workshops (n = 10), and LPG fueled vans (n = 10); totaling 56 sites.

As suggested in the vendor supplied user manual, the meter was warmed up for five minutes to get stable reading. The meter was then installed in printing press (n = 10), furniture industry (n = 10), metal workshop (n = 10), motor workshop (n = 10), newly painted buildings (n = 6), and LPG fueled vans (n = 10) of Kathmandu Valley. The sampling locations are shown in Figure 1. The sensor was installed in each indoor sites and ten data at an interval of around 5–6 min were registered by moving the sensor in different micro-environments or areas within a site. To ensure that the air diffusion in the both sensors is unobstructed, the meter was held vertically in each locations. Measurement was also made in the immediate surroundings (oudoor) of each indoor site on the same day. The time difference between the end and beginning of indoor and outdoor measurements for each sites was not more than 15 minutes. All indoor and outdoor data were collected in the time interval of 11–2:30 pm. For outdoor measurement, the meter was held around 4 m high and around ∼5–10 m away from the indoor data collection site. Also, humidity and temperature at each locations was measured with a humidity meter.

### 2.3 Data analysis

The TVOC and HCHO data were analyzed in Microsoft Excel spreadsheet to get basic statistical parameters such as mean, average and standard deviation, and percentiles. The TVOC data were also projected for 24 h averaging period using literature provided atmospheric stability formula [31]. Paired t-test was conducted in OriginPro (OriginLab Corporation, USA) between the data collected at different sites and types to know if the observed in concentration is significant. The data collected in the immediate environment (outdoor) were also analyzed and compared with the indoor environments. For comparison the indoor/outdoor ratio (I/O ratio) was also calculated, and the measured concentration was compared with WHO recommended safe limit.

## 3 Results and discussion

### 3.1 Survey study

**Figure 2:**
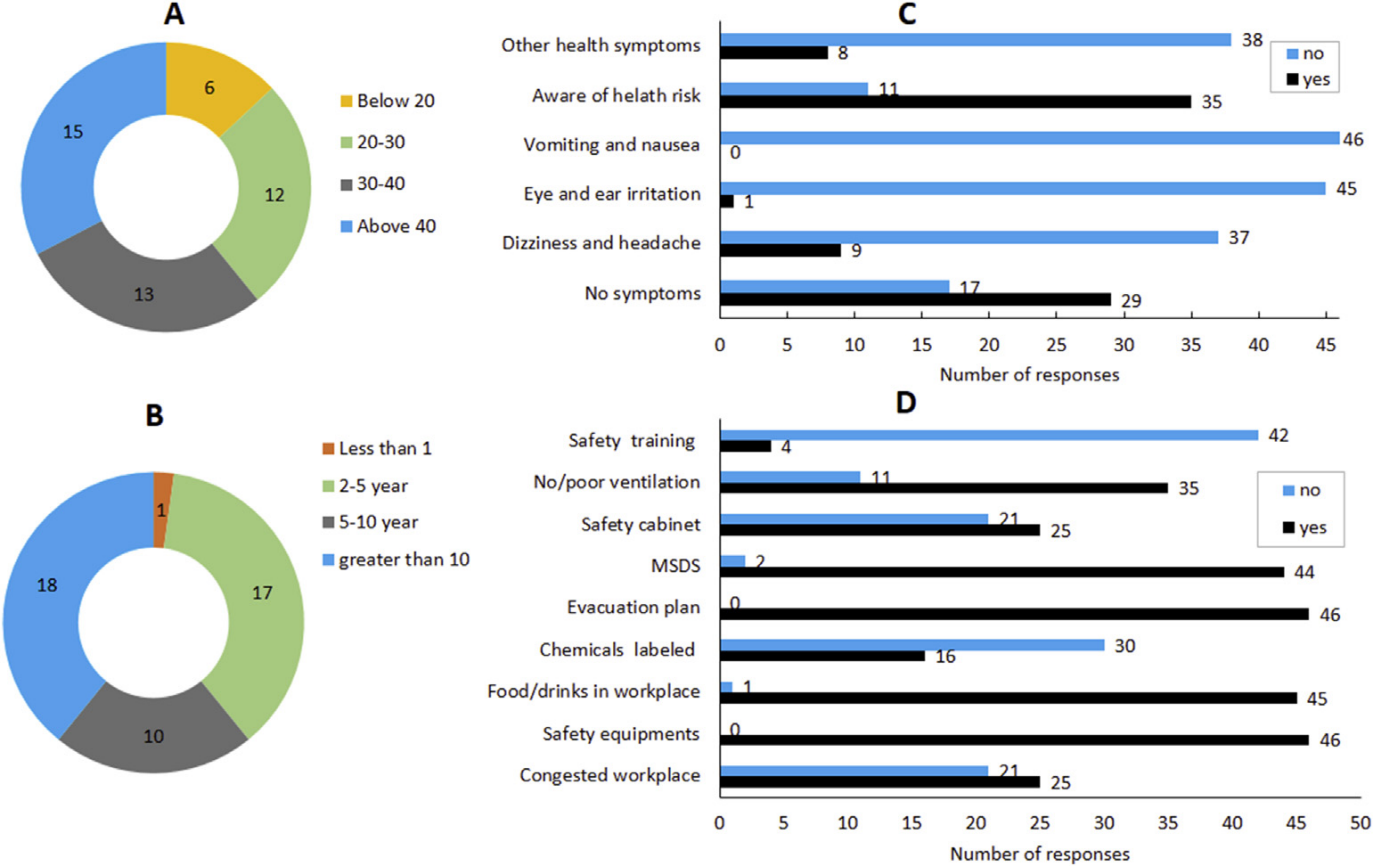
Survey study. (A) Age group distribution of the employees in the workplace. (B) Work experience. (C) Work related safety awareness and health issues. (D) Safety practices in the workplaces. In all cases, n = 46.

Survey study was conducted to get basic information on employment history, safety practices, and work related health issues in the workplace. Findings of the survey study are reported in Figure 2A‒D.

The employees' age ranged from <20 years to >40 years (Figure 2A) and have working experience ranging from less than one year (∼2%, 1/46) to more than ten years (∼22%, 10/46) (Figure 2B). We also collected response related to health awareness and health related issues. Around ∼76% (35/46) of the respondents agreed that they are aware of potential health risk in the workplace (Figure 2C). Interestingly, ∼39% of the employees (18/46) reported that have experienced some sort of health issue such as dizziness and headache (9/46), eye and ear irritation (1/46), and other (undefined) symptoms (8/46).

We also collected the responses related to the workplace safety and chemical hygiene. Only ∼9% (4/46) responded that they received chemical related safety training (Figure 2D), ∼24% workplaces (11/46) have no or poor ventilation and ∼46% workplaces (21/46) have too congested place to work. We further found that 100% of the workplace have missing evacuation plan and safety equipments, MSDS is found to be missing in only ∼4% workplaces (2/46), safety cabinet was missing in ∼46% workplaces (21/46), and chemicals were not labeled or not labeled properly in ∼65% (30/46) workplaces. Most interestingly, food and drinks is allowed in ∼98% of the workplaces (45/46). These observations suggested the poor chemical hygiene in the workplaces. Similar conclusion was reached in survey study conducted in Chemistry teaching laboratories of Nepal in 2017 [32]. The reported health issues in this study (Figure 2C), might be due to an elevated level of VOCs and poor safety standard in the workplaces.

### 3.2 Measurement of TVOC

**Figure 3:**
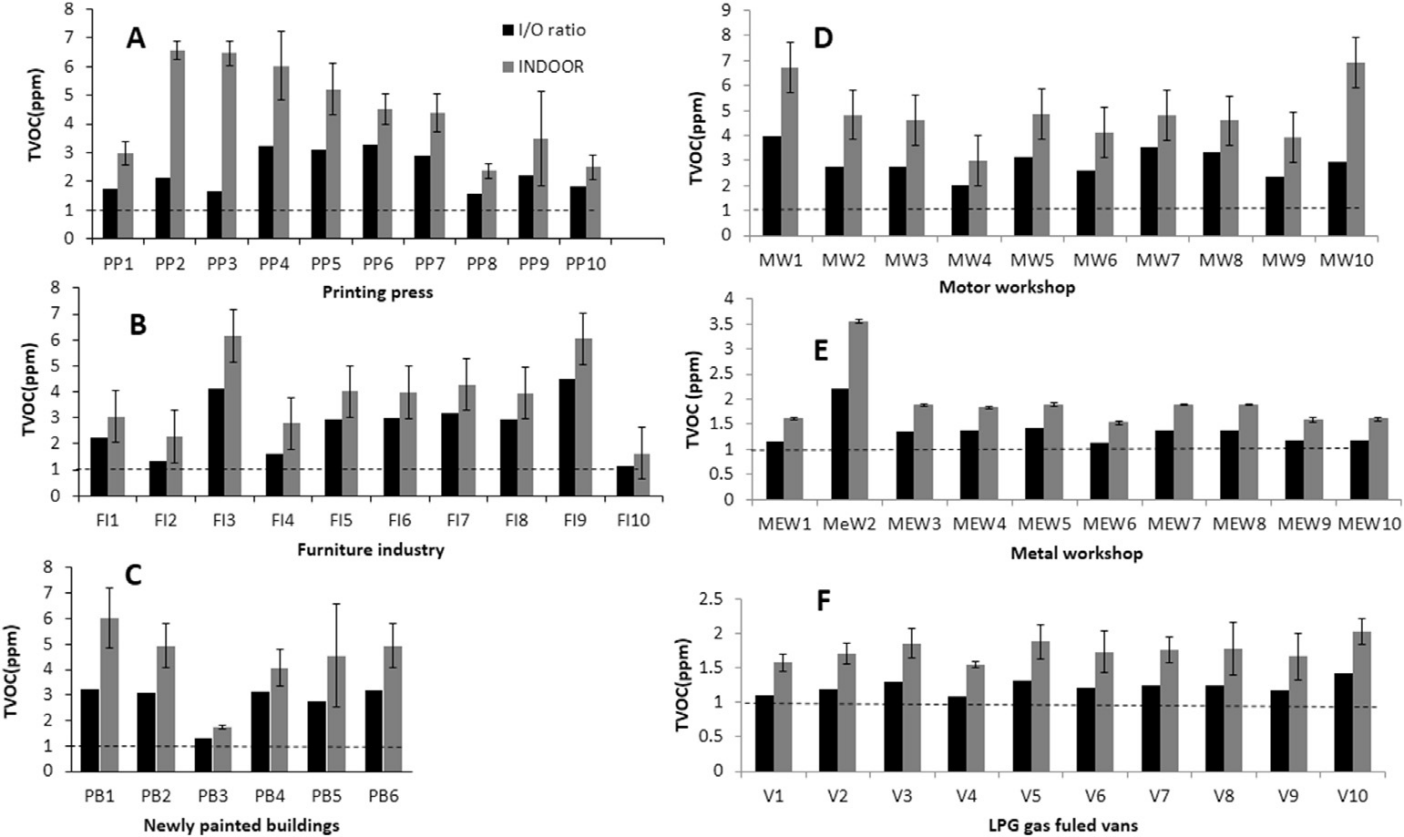
Concentration of TVOC in different workplaces (indoor) and the indoor/outdoor (I/O) ratio. (A) Printing press (PP1‒10). (B) Furniture industry (FI1‒10). (C) Newly painted buildings (PB1‒6). (D) Motor workshops (MW1‒10). (E) Metal workshops (MeW1-10). (F) LPG gas fueled vans (V1-10). The dotted line at I/O ratio of 1 is to guide the eyes.

A plot of mean TVOC concentration of different types of workplaces (indoor) is provided in Figure 3A‒F. For comparison, indoor/outdoor (I/O ratio) is also plotted in all figures. In the figures, the indoor TVOC value represent the average of ten individual data (n = 10) measured at different spots within a workplace within one hour period. VOC level within a workplace depends on several parameters. So, while collecting VOC data we also noted few important information within the workplace such as, room ventilation status, probable VOC sources and approximate distance from the source. These informations are provided as foot notes in supporting tables S1‒S6.

We did not perform longer than one hour monitoring of VOC in the indoor sites. However, to get a rough estimate of VOC for longer averaging period, one hour averaging data were projected to 24‒hour using an atmospheric stability formula (Eq. (1)) [31,33].

(1) $$C_{2}=C_{1}\times {\left(\frac{t_{1}}{t_{2}}\right)}^{n}$$

where, *C*_*1*_ and *C*_*2*_ is the concentration at shorter averaging time t_1_ (here, 1 h) and longer averaging time t_2_ (here, 24 h), respectively; and n is the stability dependent exponent (∼0.2). The projected data are provided in tables S1‒S6.

In printing presses (PPs), the mean values of TVOC ranged from ∼2.4 ppm (PP8) to 6.6 (PP2) with the corresponding I/O ratio of ∼1.6 and to 2.1 (Figure 3A and table S1). The highest I/O ratio of 3.3 was observed in PP6. In all printing presses, the I/O ratio was found higher than 1.5 indicating that indoor pollution is significantly high in all PPs (p < 0.05). We found relatively high values of TVOC near VOC sources such as ink storage area, printing machine. Also, relatively higher VOC was observed in the sites having congested working place and in unventilated conditions (table S1). These observations could explain the origin of indoor VOC in the workplaces.

In furniture industries, mean values of TVOC ranged from ∼1.6 ppm (FI10) to 6 (FI3) with the corresponding I/O ratio of ∼1 and to 4 (Figure 3B and table S2). Except in FI2 and FI10, the I/O ratio was higher than 1.5 indicating that in majority of FIs (8/10) indoor pollution was significantly higher. The indoor-outdoor mean difference was found insignificant only in FI10 (p > 0.05). We found relatively high values of TVOC near painting and machine areas and the areas where chemicals such as enamel, primer, and thinner were found (table S2) suggesting these micro-environments as major probable VOC sources.

Newly painted building (PB) are the other potential high VOC areas. In PBs, mean values of TVOC ranged from ∼1.7 ppm (PB3) to 6 (PB1) with the corresponding I/O ratio of ∼1.3 and 3.2 (Figure 3C and table S3). Except PB3, the I/O ratio was higher than 1.5 indicating that in majority of FIs (5/6) I/O ratio was significantly higher (p < 0.05). We found relatively high values of TVOC near painting areas and chemical storage areas where chemicals such as enamel, paints, thinner, sprit, tarpin oil were found (table S3).

In motor shops (MW), I/O was higher than 1.5 in all sites (10/10) with highest I/O of ∼4 observed in MW1 (Figure 3D and table S4). The indoor-outdoor mean difference was significant in all MWs (p < 0.05). In the metal workshops (MeW), I/O was higher than 1.5 was found only in one site (∼2 in MeW2) (Figure 3E and table S5). The indoor-outdoor mean difference was insignificant only in MeW7 and 8 (p > 0.05).

In LPG gas fueled vans, I/O was lower than 1.5 in all sites (10/10) (Figure 3F and table S6). Aliphatic alkanes such as propane and butane are used in LPG gas. Although strong LPG gas order was found in all the vans during data collection, these gases may not be detected by the sensor used in this study due to their high oxidation potential. This may result in underestimated TVOC values in the vans. To summarize, we found TVOC level in the workplaces in the of ∼1–7 ppm. The I/O ratio was found to be significantly higher in 34 sites (∼61%, 34/56). A brief discussion on possible sources of VOCs in the indoor and outdoor air is provided in later section.

### 3.3 Comparison of TVOC with WHO recommend limit

The recommended safe limits for TVOC and individual VOC are reported in mg/m^3^ [34]. So, for easy comparison it is important to convert the 24 h projected TVCO data to mg/m^3^. The measured concentration in ppm can be converted to mg/m^3^ (normalized to 25 °C and pressure of 1.013 atm) using Eq. (2).

(2) $$Concentration\text{ ​}in\text{ ​}mg/m^{3}=concentration\text{ ​}in\text{ ​}ppm\times f$$

where, the scaling factor *f* = molecular weight in g mole^−1^/24.45. The normalized scaling factor for formaldehyde, benzene, and toluene is 1.2 (30/24.45), 3.2 (78/24.45), 3.7 (92/24.45); respectively. For, VOC mixture the scaling factor is not known unless mole fraction of each components in the air is known. We used scaling factor of 2 (a lower estimate) to get a rough concentration estimate in mg/m^3^.

**Figure 4:**
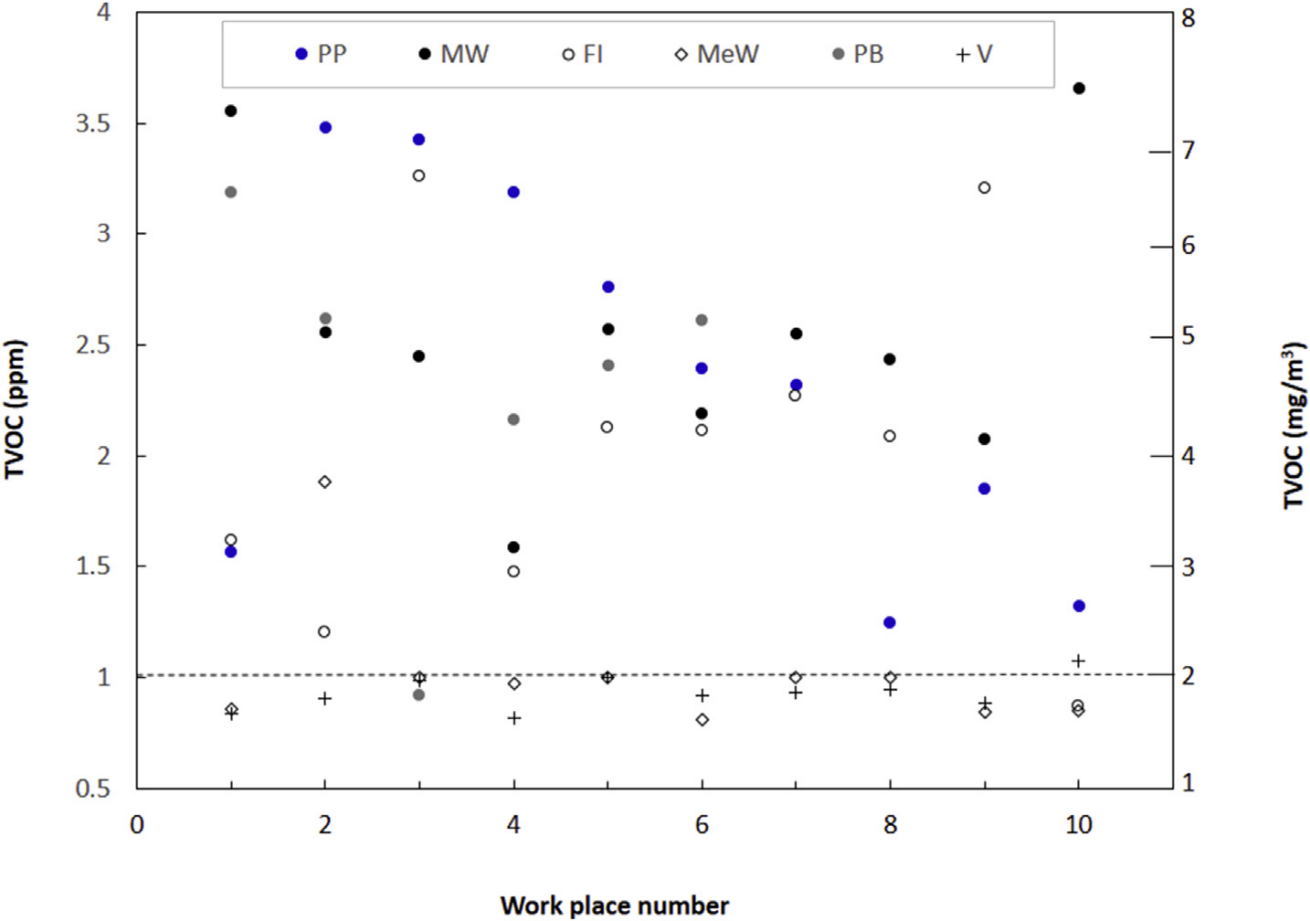
Twenty four hours projected concentration of TVOC in different indoor workplaces. PP = printing press, MW = motor workshop, FI = Furniture industry, MeW = Metal workshop, PB = newly painted buildings, and V = LPG gas fueled vans. The dotted line at 2 mg/m^3^ indicates the recommended safe limit.

The 24 h projected data in both ppm and mg/m^3^ in all indoor sites (n = 56) is provided in Figure 4. The values for all the printing presses are higher than WHO medium safe limit (∼2 mg/m^3^) with minimum and maximum values of ∼2.5 (PP8) and 7 mg/m^3^ (PP2), respectively. In motor workshops all values are higher than WHO limit and range from ∼3.2 (MW3) to 7.5 mg/m^3^ (MW10). In furniture industries and newly painted buildings all data except in FI10 and PB3 are higher than WHO limit. In metal workshop and vans except one value (MeW2 and V10) all values are lower than WHO guideline. This resulted in 36 out of 56 sites (∼64%) TVOC concentration higher than WHO safe limit. Literature studies have reported the TVOC values in the non‒industrial indoor workplaces in the range of <1–10 mg/m^3^ [18,26,35,36].

The dose dependent studies on TVOC have reported that at concentration higher than 2 mg/m^3^, significant health effects such as discomfort, headache are observed [34]. The observation of high TVOC values in majority of workplaces along with poor safety standard could explain the reported health issues in the survey study (Figure 2C).

### 3.4 Measurement of HCHO and comparison with WHO limit

**Figure 5:**
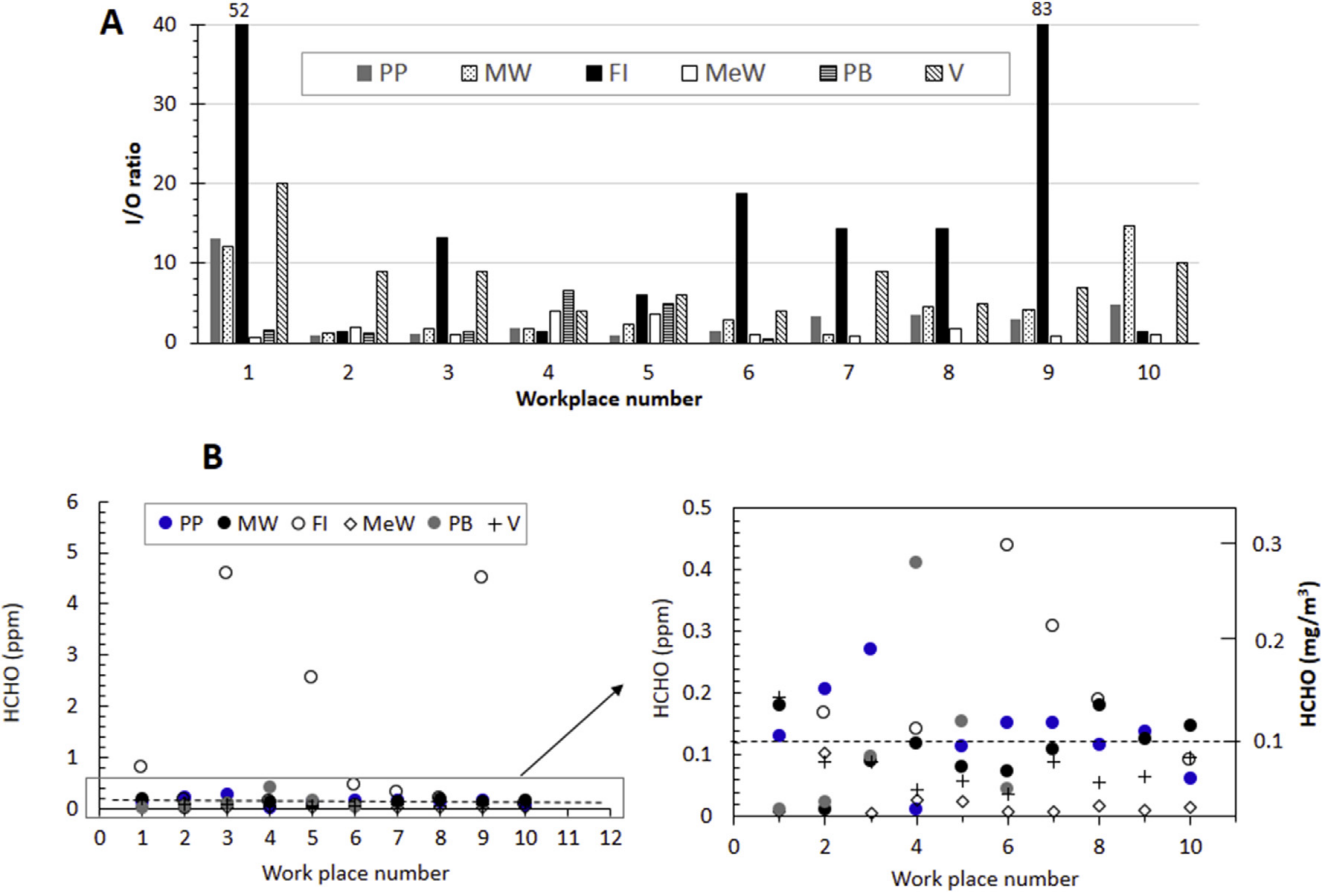
Formaldehyde data. (A) Indoor: outdoor ratio (I/O) data for all the workplaces. The high value data are truncated at ∼40. (B) Measured HCHO in different indoor workplaces. A Zoomed‒in portion of data in B is shown as inset. The dotted line at 0.1 mg/m^3^ in the inset indicates WHO recommended safe limit for short term period (30 min). PP = printed press, MW = motor workshop, FI = Furniture industry, MeW = Metal workshop, PB = newly painted buildings, and V = LPG gas fueled vans.

A plot of mean I/O formaldehyde ratio of different workplaces (total 56 sites) is provided in Figure 5A and the corresponding raw data along with 1 and 24 h projected HCHO values are provided in supporting tables S7‒12. For statistical analysis, values lower than limit of detection (LOD) (<0.01 ppm) were replaced by LOD/√2. The indoor: outdoor ratio (I/O ratio) in furniture industries, printing press, motor workshops, metal workshops, painted building, and vans was ∼1.4–83, ∼1–13, ∼1–14, ∼1–4, 1–7, and 1–20 respectively (Figure 5A). The I/O ratio was significantly higher (I/O > 1.5 and p < 0.05) in 47 sites (∼84%).

We also checked if correlation exists between TVOC and HCHO concentration in each sites. A strong positive correlation was found in furniture industry (R = 0.91) and metal workshops (R = 0.98), weak correlation in printing press (R = 0.56) and motor workshops (R = 0.58), weak negative correlation in painted building (R = -0.52), and no correlation in vans.

The WHO recommended safe limit for HCHO for short term exposure is 0.1 mg/m^3^ [37]. Formaldehyde concentration was found to be higher than WHO guidelines in 9 furniture industries (except in FI10), 6 printing presses (PP1, 2, 3, 6, 7, and 9), 2 newly painted buildings (PB4 and 5), and 1 van (V1); totaling 18 out of 56 (∼32%). In rest of the sites (44/56), HCHO concentration was found below WHO limit (Figure 5B and inset). The highest HCHO concentration was found in furniture industries with range of 0.1–4.6 (0.12–5.5 mg/m^3^), and 25^th^ and 90^th^ percentile values of 0.17 ppm (0.2 mg/m^3^) and 4.5 ppm (5.4 mg/m^3^), respectively. In all workplaces, significantly higher values of HCHO was found near the VOC sources (tables S7‒12).

Concentration‒dose dependent studies have reported that, at concentration range of 1.3–6.2 mg/m^3^, health issues such as string on eye, nose and throat and increased lacrimation and discomfort are observed. The observation of high values of HCHO in some of the sites along with poor safety standard may explain the health issues such as eye irritation and nausea experienced by the workers in the survey study (Figure 2C).

### 3.5 VOC sources and personal exposure

Source appointment studies have assigned several source categories such as industrial and vehicular emissions, residential biofuel, solvent evaporation, biofuel burning for the outdoor VOC [38]. These sources could also be the major contributor of VOC for the outdoor sites reported here. The level of indoor VOC depends on the nature of workplace, source, and safety practices. The commonly reported sources are paints, fuels, insect repellents, disinfects, building materials and others [39]. In the indoor sites studied here, we found significantly elevated VOC and formaldehyde level near point sources such as chemical storage and use areas, machines and machine parts (Tables 1-S12). These source categories along with the other commonly reported sources could contribute to the elevated level of VOC in the indoor sites.

Inhalation is one of the major routes of VOC exposure in humans. The respiratory intake of individual VOC is normally expressed in chronic daily intake (CDI). CDI (mg/day per adult body mass) depends on several parameters such as concentration individual pollutant in the workplace, exposure duration and frequency, absorption factor. Considering the absorption factor of 90%, CDI of a pollutant in a workplace can be estimated as [40,41]:

(3) $$CDI_{i}=\frac{C_{i}\times IR\times f\times D}{L\times Y}\times 90\%$$

where, *C*_*i*_ is time weighted concentration of *i*^th^ VOC (mg/m^3^), IR the Inhalation rate (m^3^/day), f the exposure frequency (days/year), D the exposure duration (years) i.e. working lifetime of an adult, L average life time of adult (years), and Y number of days per year (365 days).

We used inhalation rate of 19 m^3^/day [40], inhalation frequency of 281 days/year (working days excluding public holidays in Nepal), average life expectancy of 71 years [42], exposure duration of 45 years (from 18 years to retirement year of 63 years) to get a rough estimate CDI for formaldehyde. Except in FI1 (∼11 mg/day), FI3 (∼65 mg/day), FI5 (∼36 mg/day), and FI9 (∼64 mg/day) the CDI was below WHO recommend limit of ∼8 mg/day per adult body mass [34].

In the current study we implemented a commercial VOC sensor to measure concentration of TVOC and formaldehyde in indoor and outdoor air directly. In future, combination of direct and mass based measurement techniques can be used to compare the results of two techniques and explore both short and long terms variation of TVOC and most common individual VOCs. Also, a systematic study on exposure assessment of individual VOCs in the workplaces can be explored.

## 4 Conclusions

To summarize, we measured TVOC and HCHO concentration six types of workplaces (n = 56 sites) of Kathmandu Valley. A survey study was conducted to get information on the safety practices and work related health issues in the workplaces. The indoor: outdoor TVOC and HCHO ratio (I/O ratio) was found to be significantly higher (I/O > 1.5 and p < 0.05) in 34 (61%) and 47 (84%) sites, respectively. Most importantly, we found TVOC concentration higher than WHO limit (2 mg/m^3^) in ∼64 % sites and HCHO concentration higher than WHO limit (0.1 mg/m^3^) in ∼32% sites. This study suggested that indoor air quality and safety practices in significant number of indoor sites is poor and proper action(s) should be taken to minimize the concentration, VOC exposure, and short and long term health effects. The control measures could be: 1) installation of safety hood/cabinet, 2) storage of VOCs in closed containers, 3) use of personal protective equipments while handling those chemicals, 4) proper safety training for the employees. Also, a proper guideline and its effective implementation for safe use of VOCs in indoor workplaces is needed.

## Declarations

### Author contribution statement

Madhav Kharel: Performed the experiments; Analyzed and interpreted the data; Wrote the paper.

Surendra Chalise & Baburam Chalise: Performed the experiments; Wrote the paper.

Deepak Gewali: Contributed reagents, materials, analysis tools or data.

Khaga Raj Sharma: Contributed reagents, materials, analysis tools or data.

Hari Paudyal: Analyzed and interpreted the data; Contributed reagents, materials, analysis tools or data.

Bhanu Neupane: Conceived and designed the experiments; Analyzed and interpreted the data; Contributed reagents, materials, analysis tools or data; Wrote the paper.

### Funding statement

This work was supported by Ministry of Forests and Environment, Department of Environment, Government of Nepal, Nepal; Grant ID: 12522.

### Data availability statement

Data included in article/supplementary material/referenced in article.

### Declaration of interests statement

The authors declare no conflict of interest.

### Additional information

No additional information is available for this paper.
