# Supplementary material for: Assessing volatile organic compound level in selected workplaces of Kathmandu Valley
Source: Heliyon. 2021 Oct 27;7(11):e08262. doi: 10.1016/j.heliyon.2021.e08262 (PMC8571507; doi:10.1016/j.heliyon.2021.e08262)
Supplement: Supporting_information [file mmc1.docx]

Supporting information for

**Assessing volatile organic compound level in selected workplaces of Kathmandu Valley**

Madhav Kharel^1^, Surendra Chalise^1^, Baburam Chalise^1^, Khaga Raj Sharma^1^, Deepak Gyawali^1,2^ , Hari Paudyal^1^, Bhanu Bhakta Neupane^1*^

*1) Central Department of Chemistry, Tribhuvan University, Kathmandu, Nepal*

*2) Ministry of Forests and Environment, Department of Environment, Government of Nepal, Nepal*

Corresponding author*: bbneupane@cdctu.edu.np*

**Survey questionnaires**

Indoor and outdoor air pollution is one of the leading causes of death globally. The major contributors to air pollution are particulate matter (PM) and volatile organic compounds (VOCs). We are conducting a research on the topic “To study the level of volatile organic compounds in various workplaces of Kathmandu valley” in collaboration with Ministry forests and Environment Department of Environment, Nepal. This survey will attempt to collect information on the employment history, safety practices and work related health symptoms in the selected workplaces of Kathmandu valley. The questionnaires consist of total 11 questions and will take 5-10 minutes to complete. All responses will be kept anonymous.

A. Basic information

## Personal information

Name:

Age:

Sex:

Name of workplace:

Type of the workplace:

## How many years you have spent in current workplace?

B. Workplace safety

## What types of safety materials have you used while working on workshop?

- Mask
- Gloves
- Eye glass
- Apron
- others (specify)
- None of the above

## Are you aware of health risks of using chemical on workplace?

- Yes
- No

## Have you feel any of following symptoms during working with chemicals in workshop?

- Dizziness and Headache
- Eye and ear irritation
- Vomiting and nausea
- others (specify)
- None of the above

1. Have you received any (chemical related) safety related training in your workplace?

- Yes
- No

1. Do you maintain enough ventilation while working indoor?

- Yes
- NO

1. What types of chemical have you used?
2. How do you dispose these chemicals?
3. Are you aware of any (chemical related) accidents in your workplace?

- Yes
- No

If answer is yes ask its details and note it here

1. Overall observation on workplace safety (tick the following)

- Too small or congested room
- Safety equipments in the workplace

Eye wash station, Fire extinguisher, fire blanket, safety shower station

- Food or drinks in workplace close to chemicals
- Chemicals not labeled or left unattended in common room
- Evacuation plan on emergency
- MSDS
- Safety cabinet or separate room for the for the storage of chemical items

*Thank you for participating in the survey*

Lists of tables for TVOC concentration of different places

*Table S1*: The TVOC concentration in different indoor and outdoor environment of printing press.

| LOCATION | DATA | D1 | D2 | D3 | D4 | D5 | D6 | D7 | D8 | D9 | D10 | MEAN±SD^*^ | I/O | MEAN±SD^**^ |
| --- | --- | --- | --- | --- | --- | --- | --- | --- | --- | --- | --- | --- | --- | --- |
| PP1 | **O** | 1.72 | 1.74 | 1.68 | 1.71 | 1.7 | 1.65 | 1.69 | 1.7 | 1.7 | 1.65 | 1.69±0.03 | 1.75 | 0.9±0.02 |
|  | **I** | 2.12 | 3 | 3.2 | 2.85 | 2.95 | 3.03 | 3.75 | 3.1 | 2.85 | 2.75 | 2.96±0.41 |  | 1.57±0.22 |
| PP2^b,e^ | **O** | 2.85 | 3.1 | 3 | 2.8 | 2.96 | 3.14 | 3.17 | 2.75 | 3.8 | 3.65 | 3.13±0.35 | 2.10 | 1.66±.19 |
|  | **I** | 6.53 | 6.45 | 6.36 | 6.3 | 6.18 | 6.67 | 7.18 | 7 | 6.55 | 6.45 | 6.57±0.32 |  | 3.48±0.17 |
| PP3 | **O** | 3.92 | 4.12 | 4.01 | 3.9 | 3.75 | 3.87 | 3.85 | 4.12 | 3.87 | 4.16 | 3.96±0.14 | 1.63 | 2.10±0.08 |
|  | **I** | 5.57 | 6.75 | 6.56 | 6.55 | 7.23^c^ | 6.2 | 6.74 | 6.4 | 6.38 | 6.25 | 6.47±0.44 |  | 3.43±0.23 |
| PP4^d^ | **O** | 1.9 | 1.89 | 1.92 | 1.7 | 1.94 | 1.72 | 1.99 | 1.8 | 1.91 | 1.79 | 1.86±0.10 | 3.25 | 0.99±0.06 |
|  | **I** | 4.35 | 5.84 | 5.98 | 5.52 | 6.12 | 9.02^c^ | 5.52 | 5.58 | 6.32 | 6.01 | 6.03±1.19 |  | 3.20±0.63 |
| PP5^e^ | **O** | 1.69 | 1.68 | 1.7 | 1.68 | 1.82 | 1.78 | 1.68 | 1.62 | 1.59 | 1.63 | 1.69±0.07 | 3.09 | 0.90±0.04 |
|  | **I** | 5.08 | 4.36 | 5.67 | 6.35 | 5.2 | 6.5 | 5.9 | 4.3 | 3.9 | 4.85 | 5.22±0.89 |  | 2.76±0.47 |
| PP6 | **O** | 1.36 | 1.35 | 1.39 | 1.42 | 1.38 | 1.37 | 1.3 | 1.39 | 1.41 | 1.4 | 1.38±0.04 | 3.29 | 0.73±0.02 |
|  | **I** | 5.29 | 4.68 | 5.08 | 4.68 | 4.29 | 3.73 | 4.62 | 3.9 | 5 | 3.97 | 4.53±0.54 |  | 2.40±0.29 |
| PP7 | **O** | 1.56 | 1.6 | 1.58 | 1.5 | 1.64 | 1.52 | 1.48 | 1.5 | 1.46 | 1.4 | 1.53±0.08 | 2.88 | 0.81±0.04 |
|  | **I** | 4.98 | 5.2 | 4.32 | 5.6 | 3.87 | 4.29 | 3.98 | 4.32 | 3.89 | 3.39 | 4.39±0.69 |  | 2.33±0.37 |
| PP8 | **O** | 1.45 | 1.44 | 1.47 | 1.49 | 1.51 | 1.48 | 1.52 | 1.45 | 1.56 | 1.59 | 1.50±0.05 | 1.58 | 0.80±0.027 |
|  | **I** | 1.97 | 2.01 | 2.75 | 2.4 | 2.35 | 2.31 | 2.3 | 2.7 | 2.44 | 2.36 | 2.36±0.25 |  | 1.25±0.14 |
| PP9 | **O** | 1.46 | 1.63 | 1.64 | 1.65 | 1.61 | 1.62 | 1.64 | 1.42 | 1.58 | 1.49 | 1.58±0.09 | 2.22 | 0.84±0.05 |
|  | **I** | 2.46 | 2.51 | 2.47 | 2.38 | 2.48 | 6.24^c^ | 2.55 | 2.49 | 6.13 | 5.23 | 3.50±1.66 |  | 1.86±0.88 |
| PP10^a^ | **O** | 1.41 | 1.32 | 1.3 | 1.41 | 1.43 | 1.42 | 1.39 | 1.35 | 1.37 | 1.39 | 1.38±0.05 | 1.81 | 0.74±0.03 |
|  | **I** | 2.16 | 2.17 | 2.78 | 3.2 | 2.68 | 2.62 | 2.32 | 3.01 | 1.97 | 2.01 | 2.5±0.43 |  | 1.32±0.23 |

PP= printing press, D= means data taken, I/O= indoor/outdoor ratio.

*Mean±standard deviation ten measurements (~1 hour).

**Mean±standard deviation of the data 24 hour projection.

^a^well ventilated.

^b^large machines running, small and congested workplace.

^c^~1m from ink store.

^d^numbers of printing, offset press, flex print are on the same site.

^e^no ventilation, workshop are between heavy building, underground room congested workplace.

*Table S2*: The TVOC Concentration in different indoor and outdoor environment of Furniture industry.

| LOCATION | DATA | D1 | D2 | D3 | D4 | D5 | D6 | D7 | D8 | D9 | D10 | MEAN±SD^*^ | I/O | MEAN±SD^**^ |
| --- | --- | --- | --- | --- | --- | --- | --- | --- | --- | --- | --- | --- | --- | --- |
| FI1 | **O** | 1.4 | 1.38 | 1.41 | 1.39 | 1.42 | 1.37 | 1.35 | 1.35 | 1.38 | 1.42 | 1.39±0.03 | 2.21 | 0.74±0.02 |
|  | **I** | 2.59 | 2.67 | 3.35 | 2.49 | 2.95 | 2.7 | 3.94^j^ | 2.4 | 3.3 | 4.2 | 3.06±0.63 |  | 1.63±0.33 |
| FI2^a^ | **O** | 1.72 | 1.8 | 1.77 | 1.75 | 1.65 | 1.66 | 1.65 | 1.64 | 1.7 | 1.73 | 1.71±0.06 | 1.34 | 0.91±0.03 |
|  | **I** | 2.34 | 1.94 | 2.05 | 2.24 | 2.14 | 3.24^j^ | 2.44 | 2.11 | 2.01 | 2.21 | 2.28±0.38 |  | 1.21±0.20 |
| FI3 | **O** | 1.43 | 1.52 | 1.42 | 1.53 | 1.62 | 1.58 | 1.43 | 1.48 | 1.52 | 1.51 | 1.51±0.07 | 4.1 | 0.80±0.04 |
|  | **I** | 3.9 | 3.8 | 6.4 | 7.8^k^ | 6.78 | 7.28 | 6.78^k^ | 6.87 | 5.98 | 6.02 | 6.17±1.34 |  | 3.27±0.71 |
| FI4 | **O** | 1.84 | 1.89 | 1.88 | 1.7 | 1.69 | 1.67 | 1.71 | 1.6 | 1.66 | 1.7 | 1.74±0.10 | 1.61 | 0.92±0.06 |
|  | **I** | 1.98 | 2.46 | 2.45 | 2.35 | 2.64 | 2.85 | 3.01 | 3.85 | 3.3 | 2.96 | 2.79±0.54 |  | 1.48±0.03 |
| FI5 | **O** | 1.43 | 1.37 | 1.38 | 1.4 | 1.39 | 1.32 | 1.34 | 1.37 | 1.29 | 1.38 | 1.37±0.05 | 2.94 | 0.73±0.03 |
|  | **I** | 3.37 | 3.34 | 3.2 | 3.32 | 3.26 | 5.23^k^ | 3.54 | 3.62 | 4.45^j^ | 6.78^k^ | 4.02±1.17 |  | 2.13±0.62 |
| FI6 | **O** | 1.34 | 1.3 | 1.37 | 1.29 | 1.36 | 1.28 | 1.36 | 1.3 | 1.37 | 1.41 | 1.34±0.05 | 2.98 | 0.71±0.03 |
|  | **I** | 3.62 | 3.67 | 3.4 | 3.74 | 3.76 | 5.61^j^ | 4.65 | 3.88 | 3.62 | 3.92 | 3.10±0.66 |  | 2.12±0.35 |
| FI7 | **O** | 1.32 | 1.37 | 1.28 | 1.42 | 1.29 | 1.3 | 1.4 | 1.37 | 1.37 | 1.39 | 1.36±0.05 | 3.18 | 0.72±0.03 |
|  | **I** | 4.37 | 3.37 | 4.21 | 3.81 | 6.51^j^ | 4.34 | 2.7 | 3.87 | 4.65 | 5 | 4.29±1.02 |  | 2.27±0.55 |
| FI8 | **O** | 1.32 | 1.3 | 1.37 | 1.34 | 1.4 | 1.38 | 1.33 | 1.37 | 1.32 | 1.35 | 1.35±0.04 | 2.93 | 0.72±0.02 |
|  | **I** | 4.5 | 3.47 | 3.81 | 3.27 | 7.42^j^ | 4.21 | 4.31 | 2.37 | 3.31 | 2.74 | 3.95±1.40 |  | 2.09±0.75 |
| FI9 | **O** | 1.34 | 1.41 | 1.37 | 1.32 | 1.34 | 1.37 | 1.3 | 1.32 | 1.29 | 1.37 | 1.35±0.04 | 4.51 | 0.72±0.02 |
|  | **I** | 6.24 | 7.44^k^ | 5.38 | 4.98 | 6.42 | 5.34 | 6.24 | 5.82 | 7.34^k^ | 5.29 | 6.05±0.86 |  | 3.21±0.46 |
| FI10^a,h^ | **O** | 1.33 | 1.48 | 1.7 | 1.54 | 1.41 | 1.32 | 1.39 | 1.31 | 1.3 | 1.38 | 1.42±0.13 | 1.16 | 0.75±0.07 |
|  | **I** | 1.5 | 1.52 | 1.51 | 1.49 | 1.49 | 1.53 | 1.48 | 2.5^l^ | 1.89 | 1.48 | 1.64±0.33 |  | 0.87±0.18 |

FI= Furniture industry

^a^well ventilated, ^h^large space, well ventilated.

^j^~1m from painting area

^k^~1m from chemical bottles (enamel, primer, thinner etc), ^l^~1m from wood cutting machine

*Mean±standard deviation ten measurements (~1 hour).

**Mean±standard deviation of the data 24 hour projection.

*Table S3:* The TVOC concentration in different indoor and outdoor environments of newly painted buildings.

| LOCATION | DATA | D1 | D2 | D3 | D4 | D5 | D6 | D7 | D8 | D9 | D10 | MEAN±SD^*^ | I/O | MEAN±SD^**^ |
| --- | --- | --- | --- | --- | --- | --- | --- | --- | --- | --- | --- | --- | --- | --- |
| PB1 | **O** | 1.9 | 1.89 | 1.92 | 1.7 | 1.94 | 1.72 | 1.99 | 1.8 | 1.91 | 1.79 | 1.86±0.10 | 3.25 | 0.99±0.06 |
|  | **I** | 4.35^s^ | 5.84 | 5.98 | 5.52 | 6.12 | 9.02^r^ | 5.52 | 5.58 | 6.32 | 6.01 | 6.03±1.19 |  | 3.20±0.63 |
| PB2 | **O** | 1.57 | 1.5 | 1.53 | 1.64 | 1.67 | 1.63 | 1.65 | 1.64 | 1.59 | 1.6 | 1.61±0.06 | 3.09 | 0.85±0.03 |
|  | **I** | 4.35 | 4.4 | 4.3 | 4.6 | 7.34 | 5.01^s^ | 4.75 | 4.8 | 5.01 | 4.88 | 4.94±0.89 |  | 2.62±0.47 |
| PB3^a^ | **O** | 1.29 | 1.3 | 1.29 | 1.3 | 1.31 | 1.3 | 1.29 | 1.29 | 1.3 | 1.29 | 1.30±0.01 | 1.34 | 0.69±0.01 |
|  | **I** | 1.76 | 1.86 | 1.8 | 1.72 | 1.76 | 1.61 | 1.67 | 1.79 | 1.67 | 1.76 | 1.74±0.08 |  | 0.93±0.04 |
| PB4 | **O** | 1.31 | 1.3 | 1.29 | 1.27 | 1.34 | 1.29 | 1.3 | 1.24 | 1.32 | 1.29 | 1.30±0.03 | 3.15 | 0.69±0.02 |
|  | **I** | 3.56 | 4.35 | 3.86 | 4.15 | 5.87 | 3.69 | 3.52 | 4.12 | 3.32 | 4.4 | 4.09±0.73 |  | 2.17±0.39 |
| PB5 | **O** | 1.65 | 1.6 | 1.62 | 1.65 | 1.64 | 1.67 | 1.7 | 1.58 | 1.61 | 1.63 | 1.64±0.04 | 2.78 | 0.87±0.02 |
|  | **I** | 3.88 | 3.88 | 3.97 | 4.02 | 3.6 | 3.54 | 5.61^s^ | 3.56 | 9.99^r^ | 3.42 | 4.55±2.02 |  | 2.41±1.07 |
| PB6 | **O** | 1.27 | 1.58 | 1.61 | 1.67 | 1.68 | 1.54 | 1.53 | 1.51 | 1.48 | 1.53 | 1.54±0.12 | 3.20 | 0.82±0.07 |
|  | **I** | 6.42 | 3.71 | 4.22 | 4.7 | 5.01 | 5.06 | 5.29 | 6.2 | 4.34 | 4.3 | 4.93±0.87 |  | 2.61±0.46 |

PB= newly painted buildings

^a^large area

^r^~1m from the chemical storage area (enamel, paints, thinner, sprit, tarpin oil etc.).

^s^~1m from the painting area.

*Mean±standard deviation ten measurements (~1 hour).

**Mean±standard deviation of the data 24 hour projection.

*Table S4*: The TVOC concentration in different indoor and outdoor environment of Motor workshop.

| LOCATION | DATA | D1 | D2 | D3 | D4 | D5 | D6 | D7 | D8 | D9 | D10 | MEAN±SD^*^ | I/O | MEAN±SD^**^ |
| --- | --- | --- | --- | --- | --- | --- | --- | --- | --- | --- | --- | --- | --- | --- |
| MW1^e^ | **O** | 1.82 | 1.6 | 1.68 | 1.69 | 1.72 | 1.62 | 1.67 | 1.72 | 1.63 | 1.8 | 1.7±0.08 | 3.96 | 0.90±0.04 |
|  | **I** | 9.99^g^ | 5.27 | 5.18 | 6.21 | 6 | 5.98 | 6.72 | 8.21 | 7.28 | 6.24 | 6.71±1.47 |  | 3.56±0.78 |
| MW2 | **O** | 1.81 | 1.76 | 1.74 | 1.8 | 1.79 | 1.72 | 1.73 | 1.7 | 1.74 | 1.72 | 1.76±0.04 | 2.76 | 0.93±0.02 |
|  | **I** | 5.47 | 3.85 | 4.57 | 4.36 | 3.96 | 4.85 | 4.15 | 4.25 | 4.03 | 8.85 | 4.84±1.50 |  | 2.57±0.79 |
| MW3 | **O** | 1.75 | 1.7 | 1.63 | 1.6 | 1.67 | 1.63 | 1.73 | 1.7 | 1.69 | 1.63 | 1.68±0.05 | 2.76 | 0.89±0.03 |
|  | **I** | 4.35 | 4.3 | 4.75 | 4.22 | 4.73 | 6.64 | 4.35 | 4 | 4.34 | 4.56 | 4.63±0.75 |  | 2.45±0.40 |
| MW4^h^ | **O** | 1.52 | 1.42 | 1.47 | 1.46 | 1.49 | 1.5 | 1.48 | 1.53 | 1.5 | 1.51 | 1.49±0.04 | 2.01 | 0.79±0.02 |
|  | **I** | 2.75 | 2.65 | 2.7 | 2.35 | 2.74 | 3.76^g^ | 2.86 | 2.98 | 3.62 | 3.52 | 2.10±0.48 |  | 1.59±0.26 |
| MW5 | **O** | 1.5 | 1.53 | 1.49 | 1.48 | 1.55 | 1.53 | 1.6 | 1.58 | 1.54 | 1.55 | 1.54±0.04 | 3.16 | 0.82±0.03 |
|  | **I** | 4.56 | 3.65 | 3.7 | 4.05 | 3.63 | 4.66 | 7.34 | 5.03 | 6.1 | 5.84 | 4.86±1.24 |  | 2.58±0.66 |
| MW6 | **O** | 1.61 | 1.57 | 1.67 | 1.53 | 1.48 | 1.49 | 1.5 | 1.67 | 1.64 | 1.6 | 1.58±0.08 | 2.62 | 0.84±0.04 |
|  | **I** | 1.78 | 3.73 | 5.32 | 3.72 | 6.32^g^ | 4.32 | 2.34 | 6.02 | 4.32 | 3.45 | 4.14±1.47 |  | 2.19±0.78 |
| MW7 | **O** | 1.32 | 1.3 | 1.37 | 1.4 | 1.4 | 1.36 | 1.42 | 1.36 | 1.38 | 1.35 | 1.37±0.04 | 3.53 | 0.73±0.02 |
|  | **I** | 4.24 | 4.24 | 4.53 | 5.24 | 6.63^g^ | 3.29 | 3.72 | 4.92 | 5.25 | 6.1 | 4.82±1.04 |  | 2.56±0.55 |
| MW8 | **O** | 1.37 | 1.3 | 1.38 | 1.42 | 1.32 | 1.37 | 1.41 | 1.36 | 1.4 | 1.35 | 1.37±0.04 | 3.36 | 0.73±0.03 |
|  | **I** | 5.23 | 3.98 | 4.21 | 3.82 | 5.38 | 3.97 | 4.23 | 5.27 | 4.64 | 5.23 | 4.60±0.63 |  | 2.44±0.04 |
| MW9 | **O** | 1.54 | 1.83 | 1.62 | 1.61 | 1.71 | 1.67 | 1.54 | 1.68 | 1.58 | 1.66 | 1.65±0.09 | 2.38 | 0.88±0.05 |
|  | **I** | 2.98 | 3.4 | 4.78 | 3.38 | 5.23 | 3.39 | 4.34 | 5.01 | 2.89 | 3.74 | 3.92±0.86 |  | 2.08±0.46 |
| MW10^e,i^ | **O** | 1.99 | 1.84 | 2.09 | 2.82 | 1.89 | 2.01 | 1.95 | 2.84 | 2.95 | 2.99 | 2.34±0.50 | 2.95 | 1.24±0.27 |
|  | **I** | 5.74 | 6.02 | 6.87 | 5.95 | 6.28^f^ | 4.33 | 7.82 | 9.21 | 9.99^g^ | 6.84 | 6.91±1.70 |  | 3.66±0.90 |

MW=Motor workshop

^e^no ventilation, workshop are between heavy building, underground room congested workplace.

^f^~1m from bike repairing area

^g^~1m away from the chemical waste storage.

^h^large space, well ventilated.

^i^petrol pump ~5m from the workplace.

*Mean±standard deviation ten measurements (~1 hour).

**Mean±standard deviation of the data 24 hour projection.

*Table S5*: The TVOC concentration in different indoor and outdoor environments of Metal workshop.

| LOCATION | DATA | D1 | D2 | D3 | D4 | D5 | D6 | D7 | D8 | D9 | D10 | MEAN±SD^*^ | I/O | MEAN±SD^**^ |
| --- | --- | --- | --- | --- | --- | --- | --- | --- | --- | --- | --- | --- | --- | --- |
| MeW1^a,h,m^ | **O** | 1.4 | 1.42 | 1.41 | 1.43 | 1.4 | 1.38 | 1.37 | 1.4 | 1.37 | 1.4 | 1.40±0.02 | 1.16 | 0.75±0.02 |
|  | **I** | 1.6 | 1.65 | 1.62 | 1.67 | 1.62 | 1.6 | 1.6 | 1.63 | 1.55 | 1.6 | 1.62±0.04 |  | 0.86±0.02 |
| MeW2^a^ | **O** | 1.68 | 1.6 | 1.52 | 1.58 | 1.64 | 1.6 | 1.62 | 1.6 | 1.57 | 1.63 | 1.61±0.05 | 2.22 | 0.85±0.03 |
|  | **I** | 2.35 | 3.4 | 3.52 | 3.67 | 4.45^n^ | 4.4 | 3.25 | 3.62 | 3.52^n^ | 3.32 | 3.55±0.59 |  | 1.89±0.32 |
| MeW3 | **O** | 1.42 | 1.4 | 1.45 | 1.37 | 1.39 | 1.37 | 1.43 | 1.36 | 1.44 | 1.38 | 1.41±0.04 | 1.35 | 0.75±0.02 |
|  | **I** | 1.62 | 1.53 | 1.47 | 2.98^k^ | 1.82 | 2.18 | 1.55 | 2.02 | 1.78 | 1.87 | 1.89±0.45 |  | 0.10±0.24 |
| MeW4 | **O** | 1.34 | 1.32 | 1.37 | 1.33 | 1.38 | 1.28 | 1.32 | 1.29 | 1.34 | 1.3 | 1.33±0.04 | 1.38 | 0.71±0.02 |
|  | **I** | 1.71 | 1.64 | 1.55 | 1.82 | 3.01 | 2.32 | 1.47 | 1.54 | 1.38 | 1.87 | 1.84±0.50 |  | 0.97±0.27 |
| MeW5 | **O** | 1.3 | 1.29 | 1.36 | 1.3 | 1.34 | 1.32 | 1.35 | 1.29 | 1.36 | 1.28 | 1.40±0.04 | 1.43 | 0.70±0.02 |
|  | **I** | 1.77 | 1.73 | 1.69 | 2.98^n^ | 1.64 | 1.84 | 1.47 | 1.55 | 2.32 | 1.87 | 1.89±0.45 |  | 0.10±0.24 |
| MeW6^a,h^ | **O** | 1.41 | 1.38 | 1.39 | 1.35 | 1.36 | 1.34 | 1.3 | 1.33 | 1.32 | 1.3 | 1.35±0.04 | 1.14 | 0.72±0.02 |
|  | **I** | 1.71 | 1.62 | 1.51 | 1.61 | 1.73 | 1.39 | 1.37 | 1.4 | 1.47 | 1.51 | 1.54±0.14 |  | 0.82±0.07 |
| MeW7 | **O** | 1.38 | 1.41 | 1.42 | 1.41 | 1.39 | 1.37 | 1.38 | 1.37 | 1.35 | 1.36 | 1.39±0.03 | 1.37 | 0.74±0.02 |
|  | **I** | 1.71 | 1.67 | 1.78 | 1.62 | 1.53 | 4.31^n^ | 1.67 | 1.62 | 1.47 | 1.51 | 1.89±0.86 |  | 1.01±0.46 |
| MeW8 | **O** | 1.38 | 1.41 | 1.42 | 1.36 | 1.41 | 1.39 | 1.37 | 1.38 | 1.37 | 1.35 | 1.39±0.03 | 1.37 | 0.74±0.02 |
|  | **I** | 1.71 | 1.67 | 1.78 | 1.62 | 1.53 | 4.31 | 1.67 | 1.62 | 1.47 | 1.51 | 1.89±0.86 |  | 1.01±0.46 |
| MeW9^a,h^ | **O** | 1.46 | 1.42 | 1.38 | 1.39 | 1.37 | 1.32 | 1.3 | 1.32 | 1.37 | 1.3 | 1.37±0.06 | 1.17 | 0.73±0.03 |
|  | **I** | 1.71 | 1.72 | 1.62 | 1.63 | 1.61 | 1.52 | 1.58 | 1.62 | 1.49 | 1.39 | 1.59±0.11 |  | 0.85±0.06 |
| MeW10^a^ | **O** | 1.32 | 1.3 | 1.37 | 1.37 | 1.39 | 1.4 | 1.31 | 1.32 | 1.35 | 1.36 | 1.35±0.04 | 1.19 | 0.72±0.02 |
|  | **I** | 1.62 | 1.67 | 1.71 | 1.62 | 1.67 | 1.52 | 1.51 | 1.61 | 1.49 | 1.62 | 1.61±0.08 |  | 0.85±0.04 |

MeW= metal workshop

^a^well ventilated, ^h^large space, well ventilated.

^k^~1m from chemical bottles (enamel, primer, thinner etc).

^m^workplace is near international airport.

^n^~1m from thechemical bottles (metal primer, paints etc.).

*Mean±standard deviation ten measurements (~1 hour).

**Mean±standard deviation of the data 24 hour projection.

*Table S6:* The TVOC concentration of different indoor and outdoor environments of LPG fueled vans.

| TVOC | D1 | D2 | D3 | D4 | D5 | D6 | D7 | D8 | D9 | D10 | MEAN±SD^*^ | I/O | MEAN±SD^**^ |
| --- | --- | --- | --- | --- | --- | --- | --- | --- | --- | --- | --- | --- | --- |
| V1 | 1.73 | 1.83 | 1.63 | 1.52 | 1.5 | 1.43 | 1.51 | 1.55 | 1.63 | 1.47 | 1.58±0.13 | 1.1 | 0.84±0.67 |
| V2 | 2.07^t^ | 1.87 | 1.71 | 1.66 | 1.68 | 1.64 | 1.53 | 1.64 | 1.62^x^ | 1.63 | 1.71±0.16 | 1.2 | 0.91±0.82 |
| V3 | 1.71 | 1.72 | 1.75^x^ | 1.68 | 1.8 | 1.79 | 1.82 | 1.77 | 2.25^u^ | 2.28 | 1.86±0.22 | 1.3 | 0.99±0.12 |
| V4 | 1.58 | 1.48 | 1.52 | 1.53 | 1.58 | 1.56 | 1.58^x^ | 1.52 | 1.48 | 1.6 | 1.55±0.05 | 1.1 | 0.82±0.03 |
| V5 | 1.79 | 2.08 | 2.2 | 2.24 | 2.05 | 1.93 | 1.7 | 1.64 | 1.61 | 1.6 | 1.89±0.25 | 1.3 | 0.10±0.14 |
| V6 | 2.54 | 1.7 | 1.64 | 1.86 | 1.72 | 1.64 | 1.59 | 1.56 | 1.55 | 1.54 | 1.74±0.30 | 1.2 | 0.92±0.16 |
| V7 | 2.17 | 2 | 1.81 | 1.74 | 1.54^x^ | 1.62 | 1.68 | 1.65 | 1.64 | 1.77 | 1.77±0.20 | 1.2 | 0.94±0.11 |
| V8 | 2.57^t^ | 2.38^t^ | 1.92^x^ | 1.66 | 1.58 | 1.54 | 1.51 | 1.54 | 1.5 | 1.62 | 1.79±0.39 | 1.3 | 0.95±0.22 |
| V9 | 2.03^t^ | 2.51^t^ | 1.56 | 1.57 | 1.5 | 1.51 | 1.58 | 1.5 | 1.47 | 1.43 | 1.67±0.35 | 1.2 | 0.89±0.19 |
| V10 | 2.2 | 2.29^u^ | 2.03 | 2 | 2.12 | 2.15 | 2.17^t^ | 1.78 | 1.76 | 1.79 | 2.03±0.20 | 1.4 | 1.08±0.11 |
| O^w^ | 1.42 | 1.43 | 1.45 | 1.39 | 1.41 | 1.46 | 1.4 | 1.48 | 1.42 | 1.43 | 1.43±0.03 |  | 0.76±0.02 |
| N^v^ | 1.74 | 1.78 | 1.77 | 1.84 | 1.65 | 1.7 | 1.81 | 1.91 | 1.7 | 1.69 | 1.76±0.08 |  | 0.94±0.05 |

V=Van

^t^pick up time (more gas used up by van and high emission).

^u^Near gas cylinder.

^v^the data are taken when the van was at rest.

^w^outdoor data of van stand.

^x^inside the van (door is closed, glass windows were partially opened).

*Mean±standard deviation ten measurements (~1 hour).

**Mean±standard deviation of the data 24 hour projection.

**Lists of tables for HCHO concentration of different places**

*Table S7:* The HCHO concentration of different indoor and outdoor environments of printing press.

| HCHO | DATA | D1 | D2 | D3 | D4 | D5 | D6 | D7 | D8 | D9 | D10 | MEAN^*^ | I/O | MEAN^**^ |
| --- | --- | --- | --- | --- | --- | --- | --- | --- | --- | --- | --- | --- | --- | --- |
| PP1 | **O** | n.d | 0.01 | 0.01 | n.d | n.d | n.d | 0.01 | n.d | n.d | n.d | 0.01 | 13.1 | 0.005 |
|  | **I** | 0.16 | 0.12 | 0.1 | 0.15 | 0.18 | 0.12 | 0.12 | 0.1 | 0.15 | 0.11 | 0.13 |  | 0.069 |
| PP2 | **O** | 0.24 | 0.22 | 0.2 | 0.22 | 0.2 | 0.26 | 0.24 | 0.23 | 0.23 | 0.24 | 0.23 | 0.9 | 0.121 |
|  | **I** | 0.2 | 0.17 | 0.18 | 0.18 | 0.18 | 0.22 | 0.26 | 0.24 | 0.23 | 0.2 | 0.21 |  | 0.109 |
| PP3^b^ | **O** | 0.22 | 0.23 | 0.23 | 0.2 | 0.22 | 0.24 | 0.22 | 0.18 | 0.24 | 0.23 | 0.22 | 1.2 | 0.117 |
|  | **I** | 0.27 | 0.26 | 0.28 | 0.28 | 0.26 | 0.29^c^ | 0.25 | 0.27 | 0.28 | 0.28 | 0.27 |  | 0.144 |
| PP4 | **O** | n.d | n.d | 0.01 | n.d | n.d | n.d | n.d | 0.01 | n.d | n.d | 0.01 | 1.8 | 0.005 |
|  | **I** | n.d | n.d | n.d | 0.01 | 0.01 | 0.04 | n.d | n.d | n.d | 0.01 | 0.02 |  | 0.009 |
| PP5^a^ | **O** | 0.13 | 0.12 | 0.11 | 0.14 | 0.12 | 0.1 | 0.1 | 0.09 | 0.1 | 0.12 | 0.11 | 1.0 | 0.060 |
|  | **I** | 0.13 | 0.06 | 0.09 | 0.08 | 0.09 | 0.2 | 0.16 | 0.12^c^ | 0.11 | 0.1 | 0.11 |  | 0.060 |
| PP6^d^ | **O** | 0.11 | 0.12 | 0.12 | 0.09 | 0.1 | 0.08 | 0.09 | 0.13 | 0.02 | 0.14 | 0.10 | 1.5 | 0.053 |
|  | **I** | 0.16 | 0.13 | 0.17 | 0.2 | 0.09 | 0.18 | 0.11 | 0.17 | 0.14 | 0.16 | 0.15 |  | 0.080 |
| PP7^a^ | **O** | 0.06 | n.d | 0.04 | 0.08 | n.d | n.d | n.d | 0.01 | 0.08 | 0.01 | 0.05 | 3.3 | 0.025 |
|  | **I** | 0.19 | 0.18 | 0.17 | 0.14 | 0.16 | 0.11 | 0.17 | 0.12 | 0.16 | 0.13 | 0.15 |  | 0.081 |
| PP8^d^ | **O** | 0.06 | 0.03 | 0.04 | 0.03 | 0.02 | 0.04 | 0.03 | 0.03 | 0.02 | 0.03 | 0.03 | 3.5 | 0.017 |
|  | **I** | 0.17 | 0.18 | 0.22 | 0.1 | 0.11 | 0.1 | 0.09 | 0.09 | 0.06 | 0.05 | 0.12 |  | 0.062 |
| PP9^a^ | **O** | 0.11 | 0.01 | 0.04 | n.d | 0.08 | 0.08 | 0.03 | 0.01 | 0.01 | n.d | 0.05 | 3.0 | 0.024 |
|  | **I** | 0.03 | 0.19 | 0.09 | 0.14 | 0.08 | 0.17 | 0.1 | 0.19 | 0.2 | 0.18 | 0.14 |  | 0.073 |
| PP10 | **O** | n.d | n.d | n.d | 0.01 | 0.01 | 0.02 | n.d | 0.01 | n.d | n.d | 0.01 | 4.9 | 0.007 |
|  | **I** | 0.07 | 0.08 | 0.03 | 0.04 | 0.04 | 0.05 | 0.07 | 0.08 | 0.09 | 0.06 | 0.06 |  | 0.032 |

PP=Printing press, I=Indoor, O=Outdoor, D= means data taken

Concentration is ppm for all above given values.

*Mean of ten measurements (~1 hour). To calculate the mean, the n.d values (values below detection limit of 0.01 ppm) were replaced by 0.007 i.e LOD/√2.

** 24 hour projected data from the mean.

^a^ well Ventilated.

^b^congested workplace.

^c^~1m from near ink store.

^d^numbers of printing press are on the same site.

All the highest value of HCHO in the table in all printing place was measured closed to ink store or chemical store room.

*Table S8:* The HCHO concentration of different indoor and outdoor environments of Furniture industries.

| HCHO | DATA | D1 | D2 | D3 | D4 | D5 | D6 | D7 | D8 | D9 | D10 | MEAN | I/O | MEAN24 |
| --- | --- | --- | --- | --- | --- | --- | --- | --- | --- | --- | --- | --- | --- | --- |
| FI1 | **O** | n.d | n.d | 0.01 | n.d | 0.02 | n.d | n.d | n.d | n.d | n.d | 0.02 | 52.1 | 0.008 |
|  | **I** | 0.34 | 0.67 | 0.03 | 1.3 | 1.18 | 0.56 | 1.54 | 0.38 | 0.31 | 1.5 | 0.78 |  | 0.414 |
| FI2 | **O** | 0.12 | 0.16 | 0.09 | 0.13 | 0.08 | 0.12 | 0.1 | 0.14 | 0.16 | 0.12 | 0.12 | 1.4 | 0.065 |
|  | **I** | 0.09 | 0.23 | 0.12 | 0.22 | 0.18 | 0.15 | 0.2 | 0.17 | 0.14 | 0.18 | 0.17 |  | 0.089 |
| FI3^g^ | **O** | 0.2 | 0.22 | 0.25 | 0.41 | 0.46 | 0.42 | 0.41 | 0.39 | 0.32 | 0.38 | 0.35 | 13.2 | 0.183 |
|  | **I** | 4.53 | 4.23 | 3.39^i^ | 5^h^ | 4.98 | 5^h^ | 4.67 | 4.71 | 5^h^ | 4.28 | 4.58 |  | 2.425 |
| FI4^g^ | **O** | 0.06 | 0.1 | 0.09 | 0.1 | 0.08 | 0.15 | 0.09 | 0.11 | 0.15 | 0.12 | 0.11 | 1.4 | 0.056 |
|  | **I** | 0.11 | 0.1 | 0.11 | 0.17 | 0.15 | 0.18 | 0.16 | 0.18 | 0.16 | 0.1 | 0.14 |  | 0.075 |
| FI5^e^ | **O** | 0.94 | n.d | n.d | 0.31 | n.d | 0.01 | n.d | n.d | n.d | n.d | 0.42 | 6.1 | 0.222 |
|  | **I** | 1.85 | 2.12 | 1.96 | 1.9 | 2.54 | 3.12^j^ | 2.1 | 2.25 | 3.35 | 4.25 | 2.54 |  | 1.347 |
| FI6^a^ | **O** | 0.04 | 0.01 | n.d | n.d | n.d | 0.01 | 0.03 | 0.04 | n.d | 0.01 | 0.02 | 18.8 | 0.012 |
|  | **I** | 0.37 | 0.39 | 0.3 | 0.31 | 0.67 | 0.62 | 0.6 | 0.46 | 0.3 | 0.36 | 0.44 |  | 0.232 |
| FI7^a^ | **O** | 0.02 | 0.04 | 0.02 | n.d | 0.01 | 0.03 | n.d | 0.02 | 0.01 | n.d | 0.02 | 14.4 | 0.011 |
|  | **I** | 0.37 | 0.36 | 0.39 | 0.41 | 0.42 | 0.22 | 0.21 | 0.01 | 0.32 | 0.37 | 0.31 |  | 0.163 |
| FI8 | **O** | 0.02 | n.d | 0.01 | n.d | 0.01 | n.d | n.d | n.d | n.d | n.d | 0.01 | 14.3 | 0.007 |
|  | **I** | 0.11 | 0.16 | 0.17 | 0.14 | 0.51 | 0.21 | 0.02 | 0.21 | 0.2 | 0.17 | 0.19 |  | 0.101 |
| FI9^g,e^ | **O** | 0.01 | 0.02 | n.d | n.d | n.d | 0.01 | 0.02 | 0.21 | n.d | n.d | 0.05 | 83.3 | 0.029 |
|  | **I** | 5^j^ | 5 | 4.75^h^ | 5 | 5^h^ | 5 | 5^j^ | 0.21 | 5 | 5 | 4.50 |  | 2.381 |
| FI10^a^ | **O** | 0.02 | 0.03 | 0.12 | 0.01 | 0.02 | 0.02 | 0.06 | 0.21 | 0.02 | 0.12 | 0.06 | 1.4 | 0.033 |
|  | **I** | 0.1 | 0.09 | 0.04 | 0.13 | 0.1 | 0.05 | 0.01 | 0.21 | 0.16 | 0.02 | 0.09 |  | 0.048 |

FI=Furniture industry, I=Indoor, O=Outdoor, D= means data taken

*Mean of ten measurements (~1 hour). To calculate the mean, the n.d values (values below detection limit of 0.01 ppm) were replaced by 0.007 i.e LOD/√2.

** 24 hour projected data from the mean.

^a^ well Ventilated.

^e^~1m from busy public road.

^g^no ventilation, workshop are between heavy building, underground room congested workplace

^h^Formalin is found to be used in wood painting.

^i^near plywood cutting machine.

^j^near chemicals related wood buckets(thinner, enamel, chapra, glue, fevicol etc)

All the highest value of HCHO in the table in all furniture industry was measured closed to chemical or chemical store room.

*Table S9:* The HCHO concentration of different indoor and outdoor environments of Painted buildings.

| HCHO | DATA | D1 | D2 | D3 | D4 | D5 | D6 | D7 | D8 | D9 | D10 | MEAN^*^ | I/O | MEAN^**^ |
| --- | --- | --- | --- | --- | --- | --- | --- | --- | --- | --- | --- | --- | --- | --- |
| PB1^a^ | **O** | n.d | n.d | 0.01 | n.d | n.d | n.d | n.d | 0.01 | n.d | n.d | 0.01 | 1.5 | 0.004 |
|  | **I** | n.d | n.d | n.d | n.d | 0.01 | 0.01 | 0.04 | n.d | n.d | 0.01 | 0.01 |  | 0.006 |
| PB2 | **O** | n.d | n.d | 0.03 | 0.01 | n.d | 0.02 | n.d | n.d | 0.07 | 0.01 | 0.02 | 1.3 | 0.009 |
|  | **I** | 0.02 | 0.01 | n.d | 0.04 | 0.03 | n.d | 0.06 | 0.04 | n.d | 0.01 | 0.02 |  | 0.012 |
| PB3 | **O** | 0.08 | 0.07 | 0.11 | 0.09 | 0.07 | 0.04 | 0.05 | 0.08 | 0.05 | 0.06 | 0.07 | 1.4 | 0.037 |
|  | **I** | 0.08 | 0.09 | 0.07 | 0.1 | 0.12 | 0.1 | 0.09 | 0.08 | 0.12 | 0.12 | 0.10 |  | 0.051 |
| PB4^e^ | **O** | 0.27 | 0.05 | 0.01 | 0.08 | 0.09 | 0.04 | 0.02 | n.d | 0.05 | 0.02 | 0.06 | 6.5 | 0.034 |
|  | **I** | 0.51^o^ | 0.42 | 0.36 | 0.39 | 0.32 | 0.45^o^ | 0.43 | 0.38 | 0.4 | 0.46 | 0.41 |  | 0.218 |
| PB5 | **O** | 0.02 | 0.08 | 0.03 | 0.06 | n.d | 0.02 | 0.01 | 0.02 | 0.03 | 0.04 | 0.03 | 4.9 | 0.017 |
|  | **I** | 0.14 | 0.11 | 0.12 | 0.17 | 0.09 | 0.07 | 0.11 | 0.1 | 0.55^o^ | 0.09 | 0.16 |  | 0.082 |
| PB6^g^ | **O** | n.d | 0.01 | 0.02 | 0.06 | 0.07 | 1.02^o^ | n.d | 0.02 | 0.01 | 0.06 | 0.13 | 0.4 | 0.068 |
|  | **I** | 0.02 | 0.04 | 0.02 | 0.04 | 0.06 | 0.01 | 0.08 | 0.09 | 0.07 | 0.02 | 0.05 |  | 0.024 |

PB=Painted buildings, I=Indoor, O=Outdoor, D= means data taken

*Mean of ten measurements (~1 hour).

** 24 hour projected data from the mean. To calculate the mean, the n.d values (values below detection limit of 0.01 ppm) were replaced by 0.007 i.e LOD/√2.

^a^ well Ventilated.

^e^~1m from busy public road.

^g^no ventilation, workshop are between heavy building, underground room congested workplace

^o^~1m from painting materials(paints, sprits, enamel, tarpen oil etc.)

All the highest value of HCHO in the table in all painted buildings was measured closed to chemical or chemical store room.

*Table S10:* The HCHO concentration of the different indoor and outdoor environments of Motor workshops.

| HCHO | DATA | D1 | D2 | D3 | D4 | D5 | D6 | D7 | D8 | D9 | D10 | MEAN^*^ | I/O | MEAN^**^ |
| --- | --- | --- | --- | --- | --- | --- | --- | --- | --- | --- | --- | --- | --- | --- |
| MW1 | **O** | n.d | n.d | n.d | n.d | n.d | n.d | n.d | 0.01 | 0.02 | n.d | 0.02 | 12.1 | 0.008 |
|  | **I** | 0.18 | 0.17 | 0.22 | 0.23 | 0.08 | 0.18 | 0.19 | 0.2 | 0.2 | 0.16 | 0.18 |  | 0.096 |
| MW2^a^ | **O** | 0.01 | n.d | n.d | 0.01 | n.d | 0.02 | n.d | 0.01 | n.d | 0.02 | 0.01 | 1.3 | 0.007 |
|  | **I** | n.d | n.d | n.d | n.d | n.d | 0.01 | 0.03 | 0.01 | 0.02 | n.d | 0.02 |  | 0.009 |
| MW3 | **O** | 0.01 | 0.08 | 0.03 | 0.09 | 0.06 | 0.03 | 0.06 | 0.05 | 0.08 | 0.06 | 0.06 | 1.8 | 0.029 |
|  | **I** | n.d | 0.06 | 0.07 | 0.09 | 0.05 | 0.11 | 0.12 | 0.17^f^ | 0.15 | 0.08 | 0.10 |  | 0.053 |
| MW4 | **O** | 0.09 | 0.08 | 0.07 | 0.08 | 0.04 | 0.06 | 0.04 | 0.07 | 0.08 | 0.05 | 0.07 | 1.8 | 0.035 |
|  | **I** | 0.17 | 0.11 | 0.14 | 0.13 | 0.12 | 0.13 | 0.05 | 0.12 | 0.09 | 0.13 | 0.12 |  | 0.063 |
| MW5 | **O** | 0.07 | 0.01 | 0.03 | n.d | 0.01 | 0.06 | 0.03 | 0.02 | 0.05 | 0.03 | 0.03 | 2.4 | 0.018 |
|  | **I** | 0.06 | 0.07 | 0.1 | 0.11 | 0.08 | 0.07 | 0.07 | 0.09 | 0.06 | 0.1 | 0.08 |  | 0.043 |
| MW6 | **O** | n.d | 0.01 | 0.04 | 0.03 | 0.02 | 0.03 | n.d | n.d | 0.01 | 0.04 | 0.03 | 2.9 | 0.014 |
|  | **I** | 0.03 | 0.04 | 0.07 | 0.06 | 0.1 | 0.08 | 0.14 | 0.12 | 0.07 | 0.03 | 0.07 |  | 0.039 |
| MW7 | **O** | 0.22 | 0.01 | 0.24 | 0.04 | 0.02 | 0.07 | 0.04 | 0.14 | 0.16 | 0.13 | 0.11 | 1.0 | 0.057 |
|  | **I** | 0.2 | 0.17 | 0.11 | 0.09 | 0.22 | 0.03 | 0.08 | 0.07 | 0.05 | 0.08 | 0.11 |  | 0.058 |
| MW8^b^ | **O** | n.d | 0.03 | 0.04 | 0.01 | n.d | 0.07 | 0.03 | 0.06 | 0.04 | 0.04 | 0.04 | 4.5 | 0.021 |
|  | **I** | 0.22 | 0.18 | 0.2 | 0.21 | 0.17 | 0.14 | 0.18 | 0.14 | 0.17 | 0.2 | 0.18 |  | 0.096 |
| MW9^e^ | **O** | n.d | 0.02 | n.d | 0.06 | 0.03 | 0.02 | 0.04 | n.d | 0.03 | 0.01 | 0.03 | 4.2 | 0.016 |
|  | **I** | 0.09 | 0.13 | 0.14 | 0.12 | 0.17 | 0.15 | 0.16 | 0.09 | 0.12 | 0.1 | 0.13 |  | 0.067 |
| MW10^a^ | **O** | n.d | n.d | 0.01 | n.d | n.d | n.d | n.d | n.d | n.d | n.d | 0.01 | 14.8 | 0.005 |
|  | **I** | 0.11 | 0.09 | 0.22 | 0.1 | 0.17 | 0.18 | 0.16 | 0.19 | 0.14 | 0.12 | 0.15 |  | 0.078 |

MW=Motorcycle workshop, I=Indoor, O=Outdoor, D= means data taken

*Mean of ten measurements (~1 hour). To calculate the mean, the n.d values (values below detection limit of 0.01 ppm) were replaced by 0.007 i.e LOD/√2.

** 24 hour projected data from the mean.

^a^ well Ventilated.

^b^congested workplace.

^e^~1m from busy public road.

^f^~1 m from chemical storage

All the highest value of HCHO in the table in all Motorcycle workshops was measured closed to chemical or chemical store room.

*Table S11:* The HCHO concentration of different indoor and outdoor environments of Metal workshops.

| HCHO | DATA | D1 | D2 | D3 | D4 | D5 | D6 | D7 | D8 | D9 | D10 | MEAN^*^ | I/O | MEAN^**^ |
| --- | --- | --- | --- | --- | --- | --- | --- | --- | --- | --- | --- | --- | --- | --- |
| MeW1 | **O** | 0.04 | 0.01 | n.d | n.d | n.d | n.d | 0.01 | n.d | 0.01 | n.d | 0.01 | 0.7 | 0.006 |
|  | **I** | n.d | n.d | n.d | 0.01 | n.d | n.d | 0.01 | n.d | n.d | n.d | 0.01 |  | 0.004 |
| MeW2^e,g^ | **O** | 0.02 | 0.09 | 0.03 | 0.04 | 0.03 | 0.08 | 0.09^l^ | 0.06 | 0.03 | 0.07^k^ | 0.05 | 1.9 | 0.029 |
|  | **I** | 0.07 | 0.1 | 0.11 | 0.09 | 0.15 | 0.13 | 0.1 | 0.11 | 0.07 | 0.1 | 0.11 |  | 0.055 |
| MeW3^g^ | **O** | n.d | n.d | n.d | n.d | n.d | n.d | n.d | n.d | n.d | n.d | 0.01 | 1.0 | 0.004 |
|  | **I** | n.d | n.d | n.d | n.d | n.d | n.d | n.d | n.d | n.d | n.d | 0.01 |  | 0.004 |
| MeW4 | **O** | n.d | n.d | n.d | n.d | n.d | n.d | n.d | n.d | n.d | n.d | 0.01 | 3.9 | 0.004 |
|  | **I** | n.d | n.d | n.d | n.d | 0.21 | n.d | 0.01 | n.d | n.d | n.d | 0.03 |  | 0.015 |
| MeW5 | **O** | n.d | n.d | n.d | n.d | n.d | n.d | n.d | n.d | n.d | n.d | 0.01 | 3.7 | 0.004 |
|  | **I** | n.d | n.d | n.d | 0.19 | n.d | n.d | 0.01 | n.d | n.d | n.d | 0.03 |  | 0.014 |
| MeW6^a^ | **O** | n.d | 0.01 | n.d | n.d | 0.01 | n.d | n.d | n.d | n.d | 0.01 | 0.01 | 1.1 | 0.004 |
|  | **I** | n.d | n.d | n.d | n.d | 0.01 | 0.02 | n.d | n.d | 0.01 | n.d | 0.01 |  | 0.005 |
| MeW7^a^ | **O** | 0.01 | n.d | n.d | n.d | 0.01 | n.d | n.d | n.d | 0.02 | 0.03 | 0.01 | 0.8 | 0.006 |
|  | **I** | n.d | n.d | n.d | n.d | n.d | 0.02 | n.d | n.d | n.d | 0.01 | 0.01 |  | 0.005 |
| MeW8^e^ | **O** | 0.01 | n.d | n.d | n.d | 0.01 | n.d | n.d | n.d | 0.02 | 0.03 | 0.01 | 1.7 | 0.006 |
|  | **I** | n.d | n.d | n.d | n.d | n.d | 0.12^l^ | n.d | n.d | n.d | 0.01 | 0.01 |  | 0.010 |
| MeW9 | **O** | 0.01 | n.d | n.d | 0.02 | n.d | 0.01 | n.d | 0.02 | n.d | 0.04 | 0.01 | 0.9 | 0.007 |
|  | **I** | n.d | n.d | n.d | 0.01 | 0.01 | n.d | 0.02 | 0.01 | 0.03 | n.d | 0.01 |  | 0.006 |
| MeW10^a^ | **O** | 0.01 | 0.02 | n.d | 0.02 | 0.03 | 0.02 | 0.01 | n.d | n.d | 0.01 | 0.01 | 1.1 | 0.007 |
|  | **I** | n.d | n.d | 0.01 | 0.02 | n.d | 0.01 | 0.03 | 0.04 | n.d | 0.02 | 0.01 |  | 0.008 |

MeW=Metal workshop, I=Indoor, O=Outdoor, D= means data taken

*Mean of ten measurements (~1 hour). To calculate the mean, the n.d values (values below detection limit of 0.01 ppm) were replaced by 0.007 i.e. LOD/√2.

** 24 hour projected data from the mean.

^a^ well Ventilated.

^e^~1m from busy public road.

^g^no ventilation, workshop are between heavy building, underground room congested workplace

^k^ near the big welding machine.

^l^~1 from wasted engine oil store.

All the highest value of HCHO in the table in all Metal workshops was measured closed to chemical or chemical store room.

*Table S12:* The HCHO concentration of different indoor and outdoor environments of LPG fueled vans.

| HCHO | D1 | D2 | D3 | D4 | D5 | D6 | D7 | D8 | D9 | D10 | MEAN^*^ | I/O | MEAN^**^ |
| --- | --- | --- | --- | --- | --- | --- | --- | --- | --- | --- | --- | --- | --- |
| V1 | 0.29 | 0.35^p^ | 0.3 | 0.26 | 0.21 | 0.15 | 0.09 | 0.09 | 0.15 | 0.06 | 0.20 | 20 | 0.103 |
| V2^n^ | 0.09 | 0.08 | 0.1 | 0.15^p^ | 0.06 | 0.06 | 0.09 | 0.07 | 0.12 | 0.07 | 0.09 | 9 | 0.047 |
| V3 | 0.06 | 0.1 | 0.08 | 0.07 | 0.11 | 0.1 | 0.12 | 0.1 | 0.12 | 0.03 | 0.09 | 9 | 0.047 |
| V4^a^ | 0.06 | 0.05 | 0.07 | 0.02 | 0.04 | 0.03 | 0.03 | 0.07^p^ | 0.04 | 0.03 | 0.04 | 4 | 0.023 |
| V5 | 0.1 | 0.1 | 0.07 | 0.06 | 0.04 | 0.03 | 0.07 | 0.04 | 0.03 | 0.04 | 0.06 | 6 | 0.031 |
| V6 | 0.04 | 0.02 | 0.05 | 0.04 | 0.05 | 0.02 | n.d | 0.03 | 0.06 | 0.05 | 0.04 | 4 | 0.019 |
| V7 | 0.12 | 0.11 | 0.14 | 0.11^p^ | 0.09 | 0.07 | 0.09 | 0.07 | 0.06 | 0.04 | 0.09 | 9 | 0.048 |
| V8 | 0.11 | 0.09 | 0.06 | 0.08 | 0.05 | 0.04 | 0.05 | n.d | 0.04 | 0.02 | 0.05 | 5 | 0.029 |
| V9 | 0.12 | 0.1 | 0.09 | 0.06 | 0.02 | 0.03 | 0.04 | 0.09 | 0.07 | 0.04 | 0.07 | 7 | 0.035 |
| V10^n^ | 0.11 | 0.1 | 0.07 | 0.06 | 0.08 | 0.11 | 0.11 | 0.13 | 0.1 | 0.09 | 0.10 | 10 | 0.051 |
| O | 0.01 | 0.02 | 0.03 | 0.01 | n.d | 0.02 | n.d | n.d | n.d | n.d | 0.01 |  | 0.007 |
| N | 0.03 | 0.06 | 0.1 | 0.09 | 0.03 | 0.07 | 0.1 | 0.02 | 0.01 | 0.07 | 0.06 |  | 0.031 |

V= vans, O=outdoor data, N= data taken when vans were at rest.

*Mean of ten measurements (~1 hour). To calculate the mean, the n.d values (values below detection limit of 0.01 ppm) were replaced by 0.007 i.e LOD/√2.

** 24 hour projected data from the mean.

^a^ well Ventilated.

^n^door and windows were partially opened.

^p^Near gas cylinder.

All the highest value of HCHO in the table in all van was measured closed to gas cylinder.
